# Absence of nasal air flow and maxillary sinus development.

**DOI:** 10.1016/S1808-8694(15)31061-2

**Published:** 2015-10-22

**Authors:** Roberto Eustáquio dos Santos Guimarães, Celso Gonçalves Becker, Helena Maria Gonçalves Becker, Paulo Fernando Tormin Borges Crosara, Cláudia Pena Galvão, Gustavo Coelho dos Anjos

**Affiliations:** 1 Associate Professor - USP/Rib. Preto; Adjunct Professor - Department of Otorhinolaryngology, Ophthalmology and Speech and Hearing Therapy - UFMG, Full Professor - Department of Otorhinolaryngology and Ophthalmology – – UFMG.; 3 PhD, Adjunct Professor - Department of Otorhinolaryngology, Ophthalmology - UFMG.; 4 PhD, Adjunct Professor - Department of Otorhinolaryngology, Ophthalmology - UFMG.; 5 PhD. Otorhinolaryngologist.; 6 MD. Otorhinolaryngology Resident - HC-UFMG.; 7 University Hospital - Federal University of Minas Gerais.; 2 M.S. in Health Applied Social Sciences - UFMG, Otorhinolaryngologist; Resident in Facial Plastic Surgery - Clínica La Font-Bogotá. Mailing address: Gustavo Coelho dos Anjos - Rua Raul Pedreira Passos 122 São Bento Belo Horizonte MG 30350-390.

**Keywords:** choanal atresia, nasal air flow, paranasal sinuses

## Summary

Paranasal sinuses development mechanisms are not well known. Nasal air flow, according to one of the proposed theories, would be fundamental to the growth and healthy development of paranasal sinuses. **Aim:** The aim of this study was to evaluate the maxillary sinus growth and health in the presence and absence of postnasal air flow through a unique model. **Materials and methods:** Retrospective study of a series of cases; preoperative CT scans of 7 patients with unilateral choanal atresia, average age was 16.28 years (± 5.024). This study was done in a tertiary hospital, with patients treated between 1994 and 2004. The area of the maxillary sinuses was measured with the aid an Auto-Cad software. Kruskal-Wallis test was used in the statistical analysis. **Results:** Symmetrical or even bigger maxillary sinuses were found in the same side of the choanal atresia in 85.71% of the cases. There was no significant statistic difference between compared sides. CT Scan signs of sinus disease were not seem in these patients. **Conclusion:** These findings oppose the commonly accepted theory about the role of nasal air flow in health and development of paranasal cavities.

## INTRODUCTION

Choanal atresia is a rare disorder; with an incidence of 1/8,000 live births, impairing nasal airflow. Approximately 40% of choanal atresia cases are unilateral. Contrary to its bilateral counterpart, unilateral choanal atresia does not imply mandatory oral breathing and does not cause post-nasal cyclic cyanosis. For these reasons, both diagnosis and surgical treatment happen later on, sometimes at the adult age, after complete facial development. Thus, such pathology serves as a natural model for the study of the air flow role in paranasal cavity development, since it causes different aeration to a patient's right and left side paranasal cavities, allowing us to compare its effect on sinuses development.

The mechanisms responsible for paranasal cavities growth are still poorly understood^1^, ^2^, ^3^, ^4^. Some mechanisms are proposed in order to explain the phenomenon of paranasal cavity development: nasal airflow, brain growth, muscle mass traction and facial structures and, more recently, cell mechanisms (adherence and migration) ^1^.

In choanal atresia, there invariably is a secretion build up on the nasal cavity. The lack of air flow causes low oxygen pressure, reduction in cilia motility and it favors bacterial growth^5^. It was to be expected that, with all these predisposing factors, and based on the paranasal sinuses development theory, the sinuses of a person with choanal atresia should be small and sick. “A broad development of paranasal sinuses and mastoid bones tells us that during the child's growth periods they were free from diseases^6^.”

In 1927, Grove^7^, reported a case of choanal atresia in a child, and mentioned that nasal breathing is one of the most powerful factors in sinus disease prevention. Since then many authors have corroborated such statement^6^,^8^, and choanal atresia was considered a strong predisposing factor for sinus diseases.

Otacílio et al. stated that in relation to paranasal sinus development: “It's growth happens due to air pressure, occurs up to the end of adolescence, and is influenced by any type of disease affecting it.”^9^

Our goal with the present investigation is to make a comparative study of maxillary sinus development and sinus disease, in the same model (patient), in the absence and presence of posterior nasal air flow.

## MATERIALS AND METHODS

We carried out a cross-sectional study of patients with unilateral choanal atresia from 1994 to 2004 at the department of otorhinolaryngology of a tertiary care center in Brazil. We excluded those patients with history of surgical correction for choanal atresia, nasal surgery or facial trauma prior to the radiological study.

A total of 7 patients were eligible to participate. Of these, 5 were women and 2 were men. Age varied between 5 and 19 years, and 6 of them were between 17 and 19 years.

The CT scans from these patients were digitalized using a scanner and had their maxillary sinus areas measures by means of the Autocad R14 software. The CT scans were carried out in 2 to 5mm slices in the coronal and axial views. The summation of areas from the many CT slices available was compared between the choanal atresia side and the patent side, trying to achieve a percentage difference between the sizes of both maxillary sinuses of the same patient. The statistical analysis of such evaluation was carried out using the Kruskall-Wallis non-parametric method. For didactic reasons we considered as symmetrical those maxillary sinuses that presented a difference of up to 5% between the area summation of the right and left sides. This study was approved by the ethics committee of our institution under protocol # 218/03.

## RESULTS

**Figure 1:**
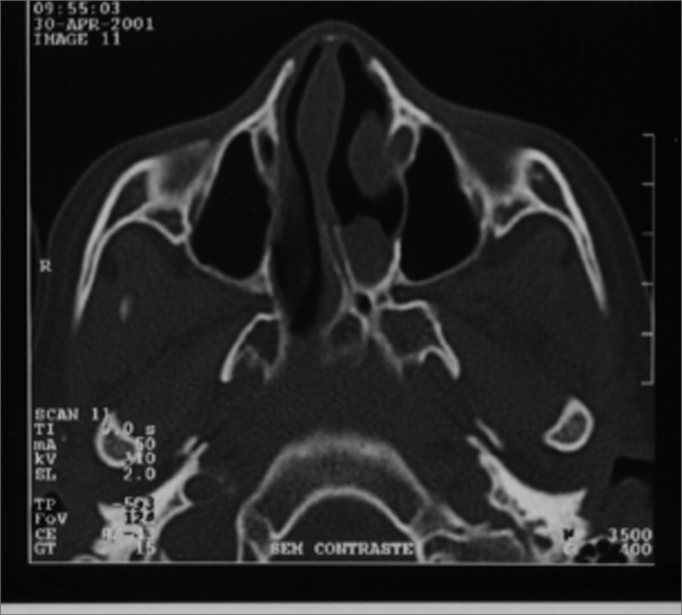
A case of left side choanal atresia and symmetrical maxillary sinuses and no sinus disease.

**Figure 2:**
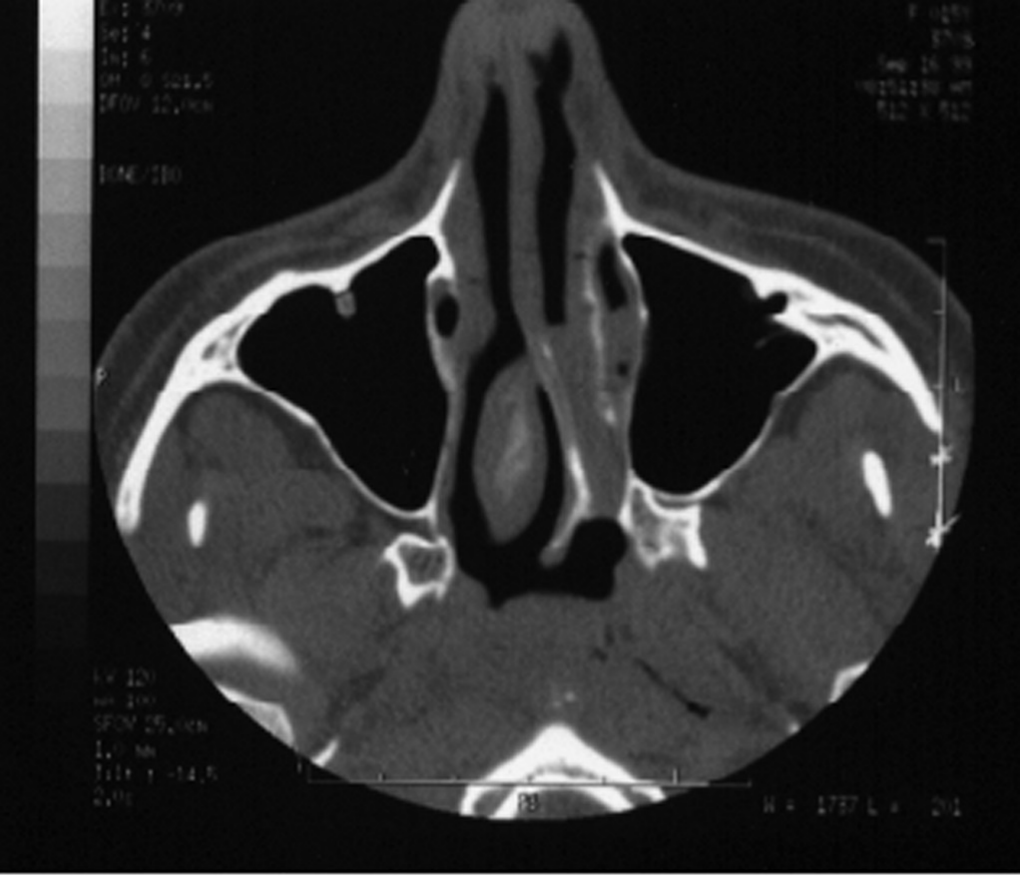
A case with CT scan characteristics similar to the one shown in Photo 1, left side choanal atresia, symmetrical maxillary sinuses and no sinus disease.

In our study we noticed maxillary sinus symmetry in 3 patients (Figures 1 and 2). In 3 other patients we noticed maxillary sinus more developed ipsilateral to the choanal atresia. One patient presented a more developed right side maxillary sinus, contralateral to the atresia. This same patient had congenital malformation on her left lower eyelid and infra-orbitary region. (Table 1)

**Table 1:** Maxillary sinuses size comparison - * Kruskal-Wallis test: Value = 4.846; p = 0.183 ** The largest size maxillary sinus was considered as 100%

	Case1	Case2	Case3	Case4	Case5	Case6	Case7
Maxillary ipsilateral to choanal atresia (%)	95,83	100	97,72	70,44	100	100	100
Maxillary contralateral to choanal atresia (%)	100	98,4	100	100	48,59	88,28	45,65

Statistical analysis with the Kruskall-Wallis method proved that there was no statistical difference between maxillary sinuses sizes in this group of patients (Value = 4.846, p = 0.183)

## DISCUSSION

We could see that of the seven patients studied, six had symmetrical maxillary sinuses, or they were even more developed on the side where there was supposedly no air flow because of choanal atresia. Such finding suggests that nasal airflow had no influence on the maxillary sinus of this side. The only patient who presented a smaller maxillary sinus in the atresia side also had facial malformations that may have influenced in the development of this sinusal cavity.10 In the seven cases, the patients did not present CT scan alterations suggesting sinus disorders, considering both, the atresic and patent sides.

This idea that nasal flow is fundamental for the development of paranasal sinuses has intrigued us for some time, especially because of the symmetry and lack of mucosa thickening in these sinuses in cases of choanal atresia. We chose the maxillary sinus as comparative parameter, because they have important growth, practically quadrupling their development from childhood to late adolescence, they are usually symmetrical and rarely absent.

A group of patients classically used as a model of association between chronic sinusitis and maxillary sinus underdevelopment are patients with cystic fibrosis. Recently, a study carried out by Kim et al.^11^ showed no difference in sinus development in children with chronic sinus disease and a control group of healthy children. Therefore, in the group of children with mucoviscidosis, the sinus development rate was statistically lower when compared to the other two groups, thus suggesting another factor in cystic fibrosis patients for this underdevelopment, other than chronic sinusitis.

Choanal atresia is frequently associated with other well described malformations, making up the so called C.H.A.R.G.E. syndrome (Coloboma of the eye; Heart disease; Atresia of the choana; Retarded growth, development or CNS anomalies; Genital hypoplasia; Ear anomalies and/or deafness)^12^. It is not uncommon to find facial, eye and central nervous system malformations associated, that may affect sinuses' development^10^.

As very well stated by Diner et al.^10^ numerous papers which relate choanal atresia with paranasal cavities underdevelopment had patients with other diseases in their series (microphthalmia, frontal lobe agenesis, maxillary osteitis) that compromised the final conclusions.

In the literature we find little evidence built by well structured methodology studies that corroborate the lack of nasal airflow causing sinusal disease. Mogensen and Tos surgically closed one of the nasal cavities in an animal model and did not observer complications or anatomical-histologic alterations in the sinusal mucosa.^13^ A recent study showed the opposite; however, the surgical closure method used in this study could have caused the alterations found.^14^

In Proetz's pioneer studies about nasal airflow, he observed that the inspiratory air flow does not reach the sinuses ostia, at least not directly, but rather the expiratory flow, already warm, moist and rich in CO_2_.^15^ Consistent histologic alterations may be found in the paranasal sinuses exposed to nasal airflow, contrary to what happens to those in whom nasal flow was interrupted, in the former we see metaplasia of the columnar cuboid epithelium.^16^

A prospective study involving 500 patients on the effect of septal deviation in the genesis of chronic rhinosinusitis also suggested that air flow alterations caused by nasal septum deviations do not bear an effective role in chronic rhinosinusitis development. In this study we did not find differences in nasal septum deviations or nasal spurs on the middle meatus between the chronic sinusitis group and patients without sinusitis. ^17^

Descriptions of choanal atresia patients bearing healthy and well developed nasal sinuses are not recent. In 1931 Stewart^18^ reported 3 cases of choanal atresia with broad bilateral sinuses development. Klossek et al.^19^ and Diner et al.^10^ showed in two series (6 and 11 patients) a symmetrical development of the paranasal sinuses in patients with characteristics similar to those of our study. Kossowska and Gasik^1^ showed a symmetrical sinuses development in patients with atresia who were operated before six months of life, in average. And, more recently, Behar and Todd20 showed in the largest series of patients so far gathered (16 cases), by using the volumetric average of the maxillary sinus, that the sinuses ipsilateral to the atresia were, in average, 0.36 ml larger than their contralateral counterparts.

## CONCLUSION

The cases hereby studied suggest that the posterior nasal airflow did not have any crucial role in the sinus development of these patients, contrary to the theory broadly accepted about paranasal cavities' development. Four other studies published in the literature corroborate these findings^10^,^16^,^17^,^18^, none of them was carried out in our country. We still require further studies, with larger series, in order to validate such statements.
